# Management of Diaphragmatic Central Tendon Plays an Important Role in the Surgical Treatment of Catamenial Pneumothorax: A Case Report

**DOI:** 10.7759/cureus.77731

**Published:** 2025-01-20

**Authors:** Daisuke Inoue, Shoji Oura

**Affiliations:** 1 Department of Surgery, Kishiwada Tokushukai Hospital, Kishiwada, JPN

**Keywords:** catamenial pneumothorax, diaphragmatic central tendon, mild pneumothorax, polyglycolic acid sheet, sex experience

## Abstract

A 25-year-old unmarried woman with sex experience was referred to our hospital for the treatment of mild pneumothorax. On pneumothorax recurrence, thoracoscopy showed no cystic lesions on the visceral pleura but small defects and slightly elevated brownish multiple lesions on the diaphragm, leading to the presumed diagnosis of catamenial pneumothorax. The patient, therefore, underwent complete resection of the diaphragmatic lesions and extensive covering of the diaphragm using a polyglycolic acid sheet with 50 mL of autologous blood application. Post-operative pathological study showed that multiple endometrial tissues resided in the diaphragm. Central tendon with endometrial tissues, but not thick diaphragm with those, had diaphragmatic defects. Immunostaining showed that both endometrial cells and stromal cells were strongly positive for estrogen receptors. The patient later underwent surgery for exacerbated pelvic endometriosis for symptom relief but has been well without catamenial pneumothorax recurrence for 21 months. Thoracic surgeons should note that catamenial pneumothorax always presents mild to moderate pneumothorax and especially needs appropriate management of the diaphragmatic central tendon when surgically treating unmarried young women with catamenial pneumothorax.

## Introduction

Endometriosis is a condition in which endometrial tissue exists ectopically outside the uterus. Like orthotopic endometrial tissue, ectopic endometrial tissue proliferates and bleeds according to the normal menstrual cycle and causes various symptoms. Therefore, ectopic endometrial tissue especially around the uterus, i.e., typical endometriosis, often causes dysmenorrhea, dyspareunia, and infertility [1].

When located in the diaphragm, ectopic endometrial tissue can cause catamenial pneumothorax. It is well known that 90% of catamenial pneumothorax cases are observed in the right thorax despite the presence of the liver [2]. The basic treatment for menstrual pneumothorax is hormone therapy, i.e., ovarian suppression therapy [3], but is sometimes limited to surgery alone, especially in young women who wish to become pregnant. We herein report the case of an unmarried young woman with catamenial pneumothorax who was successfully treated with surgery alone [4].

## Case presentation

**Figure 1:**
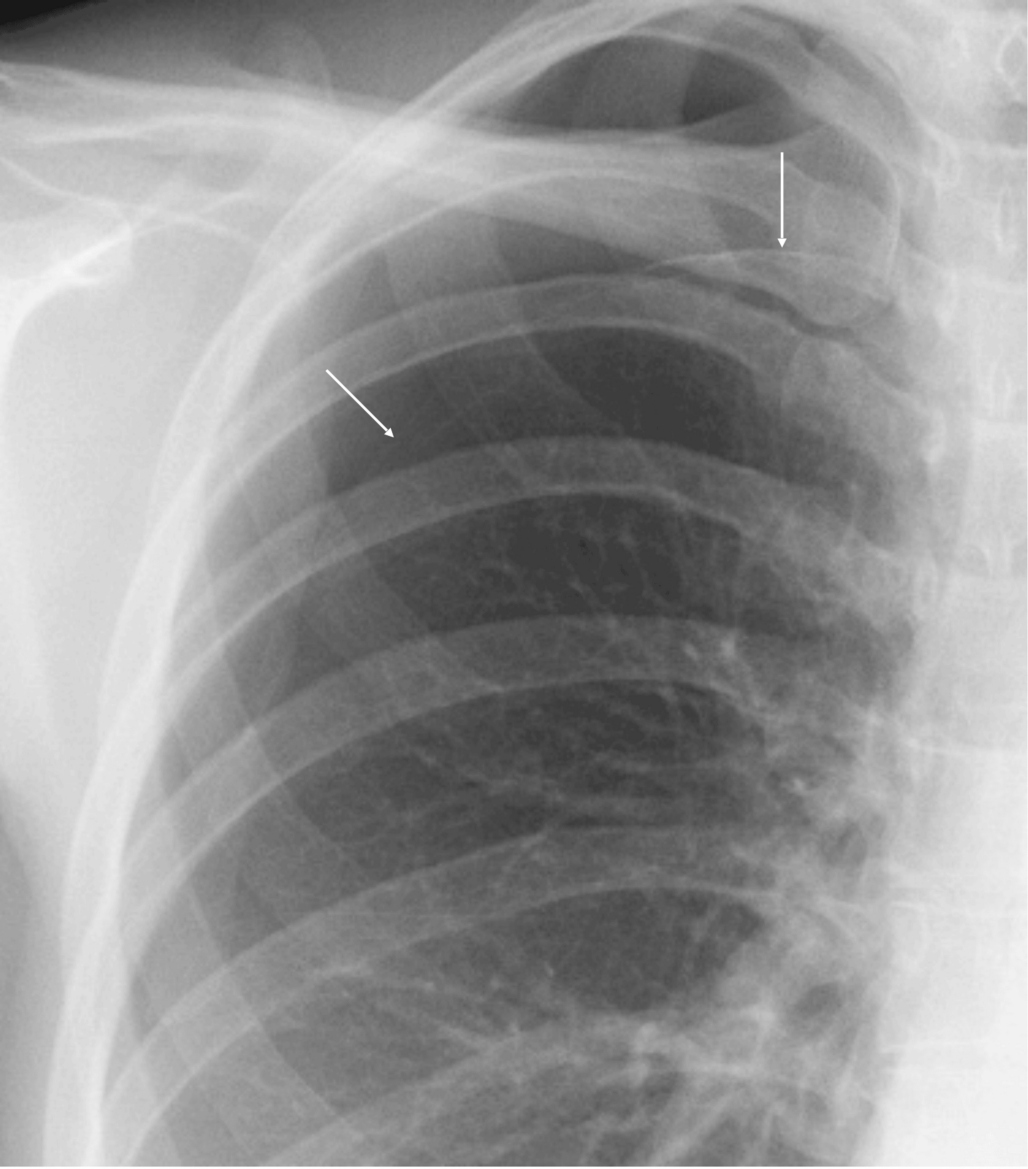
Chest radiograph showed mild collapse of the right lung.

A 25-year-old woman with long-lasting dysmenorrhea was referred to our hospital due to shortness of breath during menstruation two months before. She was unmarried but had sexual experience. A chest radiograph showed a mild right lung collapse, leading to the diagnosis of grade 1 pneumothorax (Figure 1).

**Figure 2:**
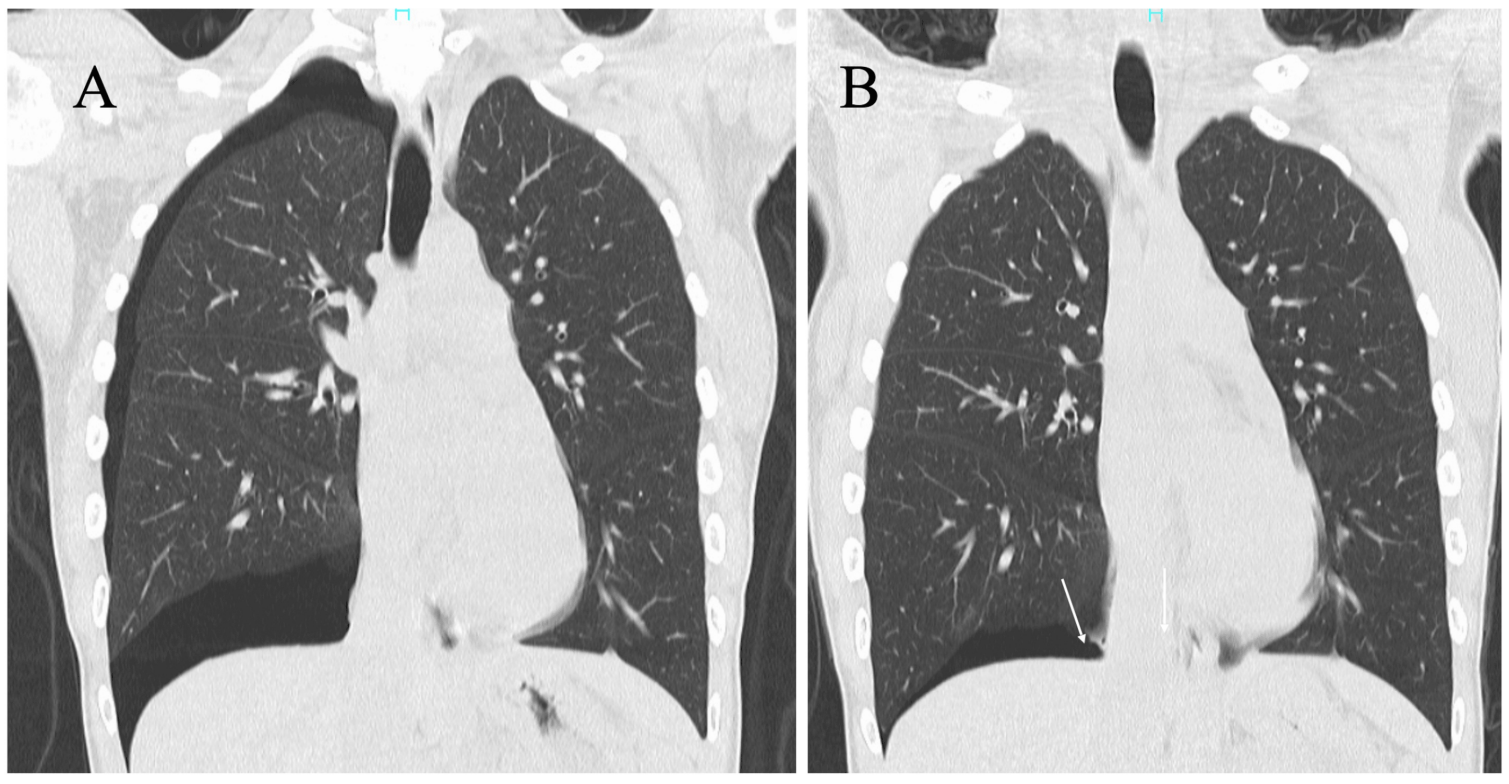
(A) Coronal computed tomography (CT) showed mild circumferential collapse of the right lung at her first visit to our hospital. (B) Coronal CT showed a small air space just above the right diaphragm at the pneumothorax recurrence.

Computed tomography (CT) showed no image-detectable bullae/blebs on the lungs in addition to the mild pneumothorax. These image findings and the characteristic symptoms led to the diagnosis of catamenial pneumothorax. The patient got symptom relief only with rest and was followed up on an outpatient basis. However, CT on dyspnea recurrence showed air leakage predominantly just above the diaphragm in the right thorax (Figures 2A, 2B).

**Figure 3:**
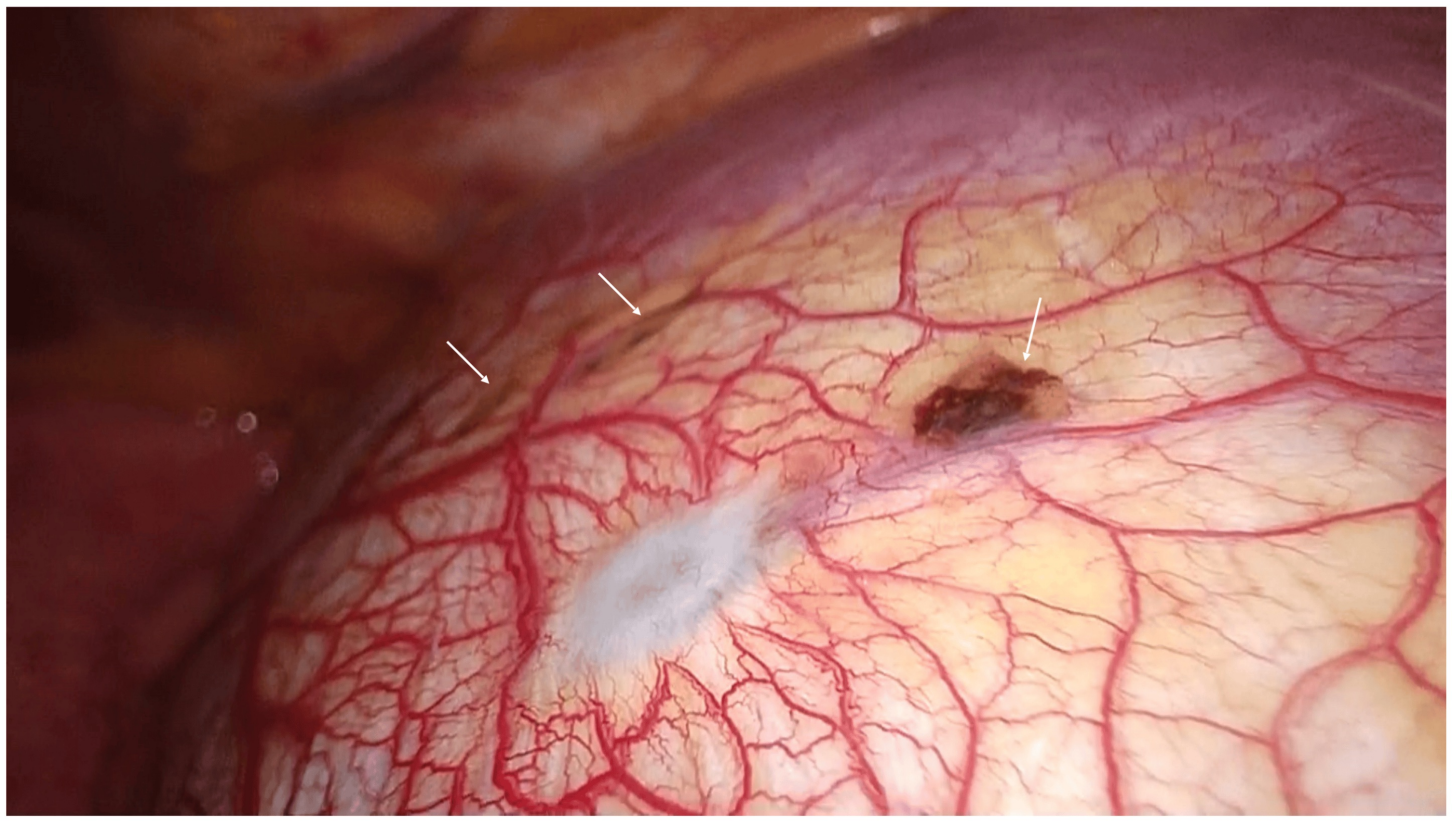
Thoracoscopic findings. Although the diaphragmatic defects were not clearly visible in this photograph, multiple brownish lesions (arrows) were observed in the central tendon.

The patient, therefore, underwent endoscopic thoracic surgery under the tentative diagnosis of recurrent catamenial pneumothorax. Thoracoscopy showed no cystic lesions on the visceral pleura but small defects and slightly elevated brownish multiple lesions on the diaphragm (Figure 3).

**Figure 4:**
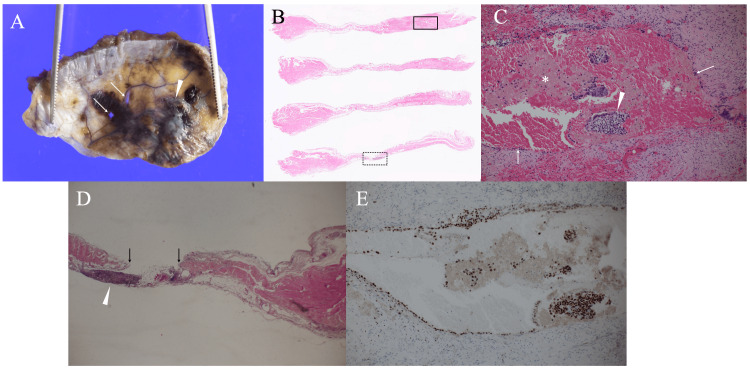
Pathological findings. (A) Small defects (arrows) and brownish nodules were observed in the resected diaphragm. (B) Serial sections of the resected specimen showed intra-diaphragmatic bleeding (square) and a diaphragmatic defect (dotted square). (C) A magnified view of the square in (B) showed that the resected diaphragm had cystically dilated endometrial cells (arrows) and stromal cells (arrowhead) with massive hemorrhage. (D) A magnified view of the dotted square in (B) showed stromal cells (arrowhead) and a defect of the diaphragmatic central tendon (arrows). (E) Both endometrial cells and stromal cells were positive for estrogen receptor.

The patient, therefore, underwent complete resection of all diaphragmatic defects and elevated lesions followed by extensive covering of the diaphragm using a polyglycolic acid sheet with 50 mL of autologous blood application. Pathological examination of the resected specimen showed that thick parts of the resected diaphragm had cystically dilated endometrial epithelium, stromal cells, and massive hemorrhage but did not have any diaphragmatic defects, whereas the central tendon had diaphragmatic defects at several small nodule sites. Immunohistochemical staining showed that both the endometrial epithelium and stromal cells were strongly positive for estrogen receptors (Figures 4A-4E).

The patient recovered uneventfully and was discharged on the fourth day after the operation. While the patient later underwent surgery for exacerbated pelvic endometriosis for symptom relief, the patient has been well without catamenial pneumothorax recurrence for 21 months.

## Discussion

Catemenial pneumothorax occurs due to the presence of ectopic endometrial tissue either on the lung or on the diaphragm. In extremely rare former cases, patients can develop moderate to severe pneumothorax due to massive air leakage directly from the lungs, often leading to large pneumothorax [5]. In the common latter cases, patients generally develop small pneumothorax [6] because negative pressure in the thorax enables air to trans-vaginally enter the thoracic cavity through the diaphragmatic defects at the ectopic endometrial tissue sites on the diaphragm. Trans-vaginal air influx to the thorax well explains the low rate of catamenial pneumothorax occurrence in teenagers with no sexual experience. The patient was in her mid-20s and unmarried but had sexual experience. Sexual experience, therefore, seems to be a prerequisite for the symptom development of catamenial pneumothorax.

It is well known that patients with Fitz-Hugh-Curtis syndrome [7] overwhelmingly have chlamydia infection around the liver. A hypothesis says that chlamydia trans-vaginally enters into the abdominal cavity and travels to the liver on the clockwise lymphatic flow in the abdominal cavity. The high incidence of catamenial pneumothorax on the right side also strongly suggests that endometrial cells migrate, like Fitz-Hugh-Curtis syndrome, to the right-side diaphragm on the lymphatic flow of the abdominal cavity.

Pathological studies showed that the diaphragmatic defects responsible for catemenial pneumothorax are located not in the thick muscle layer of the diaphragm, but in the thin diaphragmatic central tendon. These findings suggest that even if being present in the thick part of the diaphragm, endometrial cells are unlikely to cause catamenial pneumothorax. Conversely, if applied to the patients, surgery on the catamenial pneumothorax needs careful management of the diaphragmatic central tendon.

The optimal surgical techniques for catamenial pneumothorax have not yet been established [8,9]. However, a pathological study in this case revealed that endometrial tissue in the central tendon was responsible for the development of catamenial pneumothorax in most cases. It, therefore, seems important but is often clinically difficult to completely resect the diaphragmatic central tendon. Therefore, adhesion or thickening of the remaining diaphragmatic central tendon may also be useful for preventing the recurrence of catamenial pneumothorax. The polyglycolic acid sheet is an absorbable tissue reinforcement material, which is often used in thoracic surgery. The polyglycolic acid sheet has widely located fiber filaments and easily allows fibrin to reside between the fiber filaments. In this case, we used the polyglycolic acid sheet with autologous blood, instead of fibrin glue, application to enforce the residual diaphragm. In addition, absorption of the polyglycolic acid sheet generally causes inflammatory cells to infiltrate the diaphragm, making it thicken and adhere to the lung. It is naturally unclear to what extent the use of polyglycolic acid sheet contributed to preventing the recurrence of catamenial pneumothorax in this case. Diaphragmatic thickening and adhesions should be useful in preventing the recurrence of catamenial pneumothorax.

## Conclusions

Catamenial pneumothorax generally presents small pneumothorax and is often observed in young women who have a sexual experience. Conversely, young women with no sexual experience hardly develop catamenial pneumothorax symptoms due to the limited air inflow into the thorax. In addition, appropriate management of the diaphragmatic central tendon plays an important role in the surgical treatment of catamenial pneumothorax.
